# Influences of submerged plant collapse on diet composition, breadth, and overlap among four crane species at Poyang Lake, China

**DOI:** 10.1186/s12983-021-00411-2

**Published:** 2021-05-17

**Authors:** Jinjin Hou, Lei Li, Yafang Wang, Wenjuan Wang, Huiying Zhan, Nianhua Dai, Ping Lu

**Affiliations:** 1 Jiangxi Province Key Laboratory of Watershed Ecosystem Change and Biodiversity, Center for Watershed Ecology, Institute of Life Science and School of Life Science, Nanchang University, Nanchang, 330031 China; 2 Key Laboratory of Poyang Lake Environment and Resource Utilization, Ministry of Education, Nanchang University, Nanchang, 330031 China; 3 Jiangxi Poyang Lake Wetland Conservation and Restoration National Permanent Scientific Research Base, National Ecosystem Research Station of Jiangxi Poyang Lake Wetland, Nanchang, 330031 China; 4 Jiangxi Poyang Lake National Nature Reserve Authority, Nanchang, 330038 China; 5 The Institute of Biology and Resources, Jiangxi Academy of Sciences, Nanchang, 330096 China

**Keywords:** Agricultural fields, Food shortage, Hooded crane, Siberian crane, *Vallisneria* tuber, White-naped crane

## Abstract

**Background:**

Interannual variation in resource abundance has become more unpredictable, and food shortages have increasingly occurred in the recent decades. However, compared to seasonal fluctuations in resource abundance, the influences of interannual variation in resource abundance on the dietary niches of consumers remain poorly understood. Poyang Lake, China, is a very important wintering ground for the globally endangered Siberian Crane (*Leucogeranus leucogeranus*), White-naped Crane (*Grus vipio*), and Hooded Crane (*G. monacha*), as well as the non-endangered Eurasian Crane (*G. grus*). Tubers of *Vallisneria* spp., the dominant submerged macrophytes at Poyang Lake, is an important food for cranes. Nevertheless, submerged macrophytes have experienced serious degradation recently. In this study, we used metabarcoding technology to explore the consequences of *Vallisneria* tuber collapse on the diet compositions, breadths, and overlaps of the four crane species based on fecal samples collected in winter 2017 (a year with tuber collapse) and winter 2018 (a year with high tuber abundance).

**Results:**

Compared to previous studies, our study elucidates crane diets in an unprecedented level of detail. *Vallisneria* tubers was confirmed as an important food source of cranes. Surprisingly, the grassland plant *Polygonum criopolitanum* was also found to be an important food source in the feces of cranes. Agricultural fields were important foraging sites for Siberian Cranes, White-naped Cranes, and Hooded Cranes, providing foods that allowed them to survive in winters with natural food shortages. However, the three crane species preferred natural wetlands to agricultural fields when the abundance of natural foods was high. The abundance of *Vallisneria* tubers, and probably *P. criopolitanum*, greatly influenced the dietary compositions, breadths and overlap of cranes. During periods of preferred resource shortage, White-naped Cranes and Hooded Cranes widened their dietary niches, while Siberian Cranes maintained a stable niche width. The dietary niche overlap among crane species increased substantially under conditions of plentiful preferred food resources.

**Conclusions:**

Our study emphasizes the superior quality of natural wetlands compared to agricultural fields as foraging habitats for cranes. To provide safer and better foraging areas for cranes, it is urgent to restore the submerged plants at Poyang Lake. While high dietary niche overlap is often interpreted as intense interspecific competition, our study highlights the importance of taking food abundance into account.

**Supplementary Information:**

The online version contains supplementary material available at 10.1186/s12983-021-00411-2.

## Background

Interspecific competition is an important mechanism in structuring ecological communities [1, 2]. Competition may lead to resource (food, habitat, time) partitioning and facilitate the coexistence of sympatric species [1, 3, 4]. Diet partitioning has been suggested to be the primary mechanism of coexistence of many insects [5], fish [6], snakes [7], birds [8], and mammals [9].

Many ecosystems are characterized by temporal variations in resource availability, which may subsequently influence dietary niche of birds [10–12]. Migratory birds may also encounter strikingly different feeding conditions along their flyways [13, 14]. Therefore, it is critically important that birds are able to adjust their diets to explore a wide range of food resources [11, 15]. Numerous examples of dietary flexibility exist. Dunlins (*Calidris alpine*) [11], Semipalmated Sandpiper (*C. pusilla*) [16], Hooded Crane (*Grus monacha*) [17], and Black-necked Crane (*G. nigricollis*) [18] have all varied their diets with changes in prey availability. In contrast, strong dependence on a few prey types can make birds vulnerable to the reduction of prey abundance, as has been the case for Red Knot (*C. canutus*) feeding on Horseshoe Crab (*Limulus polyphemus*) eggs [19].

Optimal foraging theory is often used to predict the foraging decisions of animals [20, 21]. According to the theory, when resources are abundant, species should consume the most profitable food items with high nutrition and accessibility while ignoring less profitable foods. When preferred food items become depleted, species add less profitable foods to their diet [22, 23]. Great Knots (*C. tenuirostris*), for example, changed their diet composition and prey selection to include prey items that were physically more difficult to digest after a severe decline in food abundance and quality [24]. Optimal foraging theory predicts a reduction in dietary niche breadth during seasons of high food abundance and an increasing trend during seasons of low food abundance. This pattern of niche width variation has been observed across a range of taxa, including birds [12, 25], mammals [26, 27], fish [28, 29], and invertebrates [30, 31]. The theory also predicts an increase in dietary niche overlap between species when resources are abundant [7, 32, 33]. The increased overlap may be related to the reduced competitive interaction between species due to great resource availability. For example, there was an absence of dietary niche partitioning among shorebird species at Delaware Bay, USA, when the density of their primary prey Horseshoe Crab eggs was high [34]. Extensive degree of dietary niche overlap has also been revealed among seabird species breeding on the Argentinean Patagonian coast with superabundance of pelagic fish [35].

Understanding how dietary niche changes for any species as fluctuations in food abundance occurs is useful for designating management strategies to preserve biodiversity and ecosystem services [32, 36]. However, most research is focused on the influences of seasonal variation in food abundance on dietary niches [10, 11, 32, 37]. Due to global climate change and increased human disturbance, interannual variation in resource abundance has increased in frequency and intensity, promulgating food scarcity at higher rates in recent decades [38–40]. For example, floods that negatively affect submerged plant growth and even lead to mass death of plants have become more frequent at Poyang Lake, China [41–43], which may threaten the survival of birds that feed on them [38, 44, 45]. Seasonal variation in food abundance occurs regularly. Animals have evolved strategies to cope with the predictable environmental variation [46]. Interannual variation in food abundance occurs less frequently, although its frequency and intensity have recently increased [38–40]. Without sufficient evolutionary time, animals may not adapt well to the interannual variation; thus, it may pose a challenge for many taxa [19, 47, 48].

**Fig. 1:**
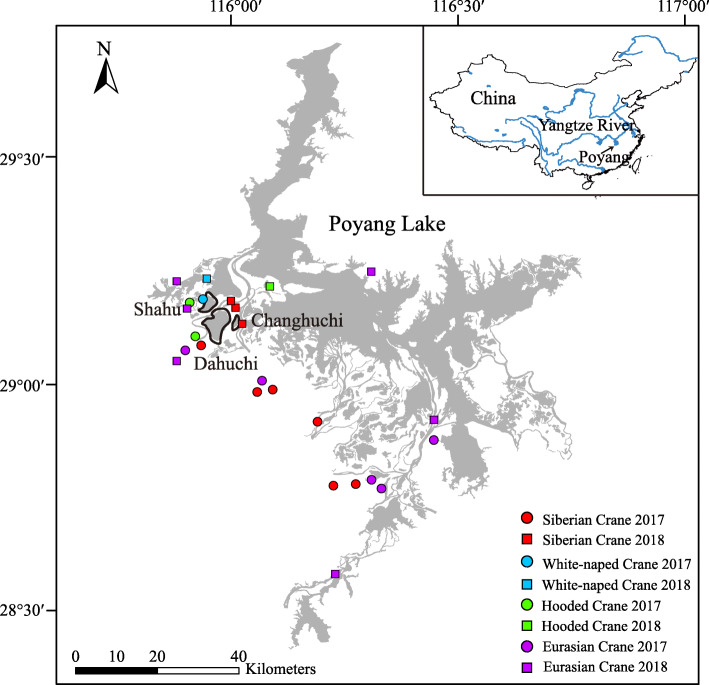
Maps showing the three *Vallisneria* tuber survey sublakes (Dahuchi, Shahu, and Changhuchi) and the sampling sites of crane feces at Poyang Lake. Circles and squares represent sampling sites in the winters of 2017 and 2018, respectively. The colors of the circles and squares correspond to the crane species: red: Siberian Crane; blue: White-naped Crane; green: Hooded Crane; and purple: Eurasian Crane. The base map shows the inundation area of Poyang Lake in winter 2008, when the water level was similar to the historical average water level

Cranes are among the most threatened families of birds [49]. Poyang Lake, which is located on the southern bank of the Yangtze River, China, is an important wintering ground for cranes in East Asia (Fig. 1). It supports approximately 98% of the estimated global population of IUCN Critically Endangered Siberian Crane (*Leucogeranus leucogeranus*), approximately 18% of the estimated global population of Vulnerable White-naped Crane (*Grus vipio*), approximately 3% of the estimated global population of Vulnerable Hooded Crane, and approximately half of the Chinese population of Eurasian Crane (*G. grus*) [50–52]. With the degradation of lakes in the middle and lower Yangtze River floodplain, Poyang Lake is playing an increasingly important role in crane protection, especially for the Siberian Crane [53, 54]. It has been suggested that other than Poyang Lake, there are no alternative wintering sites for Siberian Cranes remaining in the Yangtze River floodplain [49].

Tubers of *Vallisneria* spp., the dominant submerged macrophytes at Poyang Lake, is an important food source for cranes [38, 55, 56]. However, submerged macrophytes have degraded seriously in recent decades [57–60]. They were widely distributed throughout Poyang Lake in previous years, but were restricted to small areas in 2012 [58]. The *Vallisneria* tuber density and biomass at three sublakes (Dahuchi, Shahu and Meixihu) of Poyang Lake declined greatly in the winters from 1999 to 2017 [57, 60]. The frequency and range of *Vallisneria* tuber collapse have also increased because of frequently occurring summer flood and autumn drought, extensive aquaculture, declining water quality, and other factors [38, 57, 60].

Tuber collapse has led to dietary and foraging habitat shifts of cranes. For example, the tuber collapses in the winters of 2015 and 2016 drove thousands of Siberian Cranes, for the first time, to switch from foraging in shallow waters they typically used to paddy fields and lotus ponds [57, 61]. Their main foods changed from *Vallisneria* tubers to rice (*Oryza sativa*) seeds and lotus (*Nelumbo nucifera*) rhizomes [62]. Many White-naped Cranes, Hooded Cranes and Eurasian Cranes also moved from natural wetlands to forage in paddy fields [63, 64]. Diet shifts may influence the dietary niche width and overlap between crane species and subsequently the competition level and fitness of cranes. Given the highly endangered status of cranes and the importance of Poyang Lake in crane protection, it is important to understand the consequences of *Vallisneria* tuber collapse on diets and competition levels among crane species so that effective protection measures can be implemented.

Traditional dietary analysis methods include direct observation of foraging animals and microscopic examination of feces or gut contents. While these approaches have been suggested to be useful in some cases, they also exhibit methodological limitations [65–67]. The main drawbacks are that the methods can be inaccurate and labor-intensive [65, 68]. Recently, the emergence of DNA metabarcoding has provided new perspectives for diet analysis [65]. This method is based on amplifying and high-throughput sequencing a standardized DNA region from stomach contents or feces, and subsequently comparing it to a reference database for identification of the consumed species [65]. Compared to traditional methods, DNA metabarcoding generally provides higher taxonomic resolution, identifies more food items, and can simultaneously analyze a larger number of samples [69–71]. After its first application in assessment of the diets of Australian Fur Seals (*Arctocephalus pusillus doriferus*) [72], metabarcoding has been used successfully to study diets of herbivores [9, 73], carnivores [69, 74] and omnivores [75, 76].

In this study, we used metabarcoding technology to explore the consequences of *Vallisneria* tuber collapse on the diet compositions, niche breadths, and niche overlaps of the four crane species at Poyang Lake. Fecal samples were collected in winter 2017 (i.e., 2017/2018; a year with *Vallisneria* tuber collapse) and winter 2018 (i.e., 2018/2019; a year with high *Vallisneria* tuber abundance; see Results for details). We first explored the diets of the four crane species and their interannual variations. Most previous dietary studies of cranes were based on traditional direct observation or microscopic examination. Here, we hoped to apply the metabarcoding method to provide a more accurate and comprehensive understanding of the cranes’ diets. We then evaluated the role of agricultural fields in crane protection. *Vallisneria* tuber collapses have driven thousands of cranes to feed in agricultural fields [61, 64]. However, the proportion of domesticated species in the diets of the four crane species has not previously been quantified. Here, we determined the relative dietary importance of domesticated species in the feces of cranes. Finally, we assessed the consequences of *Vallisneria* tuber collapse on the dietary niche width and overlap among crane species. To do so, we compared the niche breadths and overlaps in winter 2017 and winter 2018 with different *Vallisneria* tuber abundances. Although we focused on cranes at Poyang Lake, our findings can provide a reference for dietary shifts of consumers in degraded wetlands worldwide.

## Results

### Variation in *Vallisneria* tuber abundance

We investigated the density and biomass of *Vallisneria* tubers at three sublakes (Dahuchi, Shahu and Changhuchi; Fig. 1) of Poyang Lake. The average tuber density and biomass were 6.68 tubers/m^2^ and 0.54 g/m^2^, respectively, in winter 2017 (Table 1). The density and biomass rapidly increased to 34.57 tubers/m^2^ and 4.11 g/m^2^ in winter 2018. The historical average tuber density and biomass at three sublakes (Dahuchi, Shahu and Meixihu) of Poyang Lake were 10.33 (± 11.27 SD) tubers/m^2^ and 3.26 (± 3.68 SD) g/m^2^ in the winters from 1999 to 2016 [57]. Tuber density in winter 2017 (t = 1.33, *P* = 0.201) and tuber biomass in winter 2018 (t = − 0.94, *P* = 0.361) did not differ from the historical average values. However, tuber biomass in winter 2017 was lower than the historical average values (t = 3.10, *P* = 0.007), and tuber density in winter 2018 was higher than the historical average values (t = − 8.88, *P* = 0.000). Therefore, winter 2017 represented a year with tuber collapse, while winter 2018 represented a year with high tuber abundance. Coinciding with the changes in tuber abundance, a total of 4014 individuals of Siberian Cranes, White-naped Cranes and Hooded Cranes were recorded in agricultural fields adjacent to Poyang Lake in winter 2017 [63], while none of the three crane species was recorded in agricultural fields in winter 2018 [77].

**Table 1:** The density and biomass of *Vallisneria* tubers at three sublakes (Dahuchi, Shahu, and Changhuchi) of Poyang Lake in the winters of 2017 and 2018

Winter	Sublake	Tuber density (tubers/m^2^)	Tuber biomass (g/m^2^)
2017	Dahuchi	9.80	0.97
	Shahu	0.28	0.02
	Changhuchi	9.97	0.62
	Average	6.68	0.54
2018	Dahuchi	6.41	0.65
	Shahu	8.97	0.60
	Changhuchi	88.32	11.07
	Average	34.57	4.11

### Collection statistics

In total, we collected 129 fecal samples, with 60 samples from winter 2017 and 69 samples from winter 2018 (Fig. 1; Additional file 1: Table S1). We collected 42 fecal samples of Siberian Cranes from 2 sublakes, 4 rice paddies, and 1 lotus pond; 20 fecal samples of White-naped Cranes from 2 sublakes and 1 rice paddy; 19 fecal samples of Hooded Cranes from 2 sublakes and 1 rice paddy; and 48 fecal samples of Eurasian Cranes from 3 sublakes and 6 rice paddies. We also collected 38 plant species commonly seen at Poyang Lake to construct a DNA barcode reference library. By downloading sequences from GenBank and sequencing by ourselves, we constructed a DNA barcode reference library containing 62 plant species.

### High-throughput DNA sequencing

**Fig. 2:**
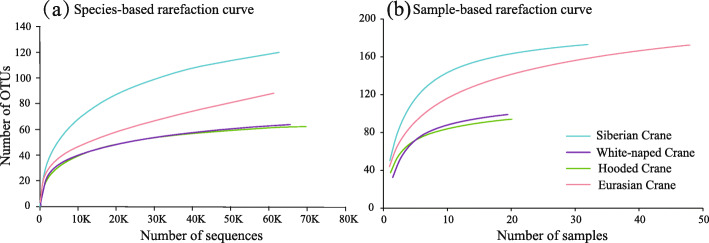
**a** Species-based and **b** sample-based rarefaction curves. The species- and sample-based rarefaction curves, built by randomly resampling sequences and samples respectively at increasing levels of accumulation, indicate whether the sequencing depth and sampling size were sufficient to characterize the diversity of dietary items. The colors of the curves correspond to the crane species: blue: Siberian Crane; purple: White-naped Crane; green: Hooded Crane; and red: Eurasian Crane

The chloroplast *trn*L intron was selected as a barcode because of its high taxonomic coverage and resolution, especially for wetland plants [70, 78]. The sequencing of the 129 fecal samples yielded a total of 9,284,819 reads. After quality filtering, 8,502,418 reads were obtained. The average number of reads per sample was 65,910 (± 26,276 SD), and the average number of operational taxonomic units (OTUs) per sample was 43 (± 15 SD). To evaluate the sequencing and sampling adequacy, individual-, species-, and sample-based rarefaction curves were built. With the sample sequencing reads increased, the individual-based rarefaction curves reached plateau in most cases (Additional file 1: Figure S1) and the species-based rarefaction curves reached plateau in the four crane species (Fig. 2a). Therefore, our sequencing depth was roughly adequate to capture the numbers of OTUs present. The sample-based rarefaction curves for each crane species reached plateau in all cases as the sampling sizes increased (Fig. 2b), suggesting that we acquired sufficient fecal samples. After comparison to the local DNA reference database, 98.99% of the total reads were assigned to specific plant taxa. Most of the reads that were not assigned to specific plant taxa had low frequency. In total, we identified 29 plant taxa, including 23 species, 4 genera, and 2 families.

### Diet compositions of the four crane species

**Fig. 3:**
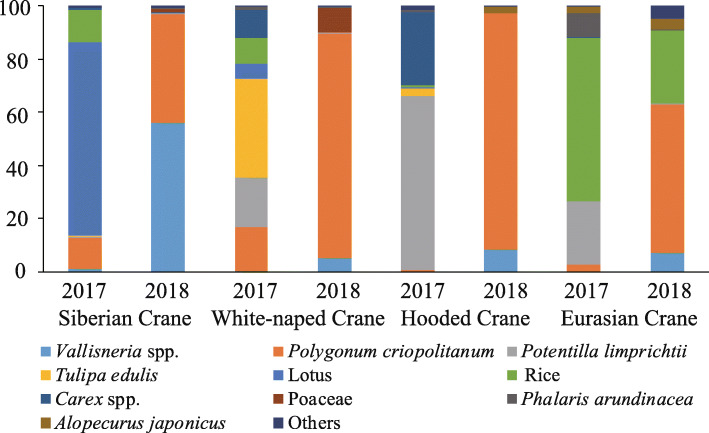
Food items in the feces of Siberian Cranes, White-naped Cranes, Hooded Cranes, and Eurasian Cranes in the winters of 2017 and 2018 at Poyang Lake

We used relative read abundance (i.e., the percentage of DNA belonging to each food item in each sample; RRA), which provides a more accurate view of species diet than frequency of occurrence (i.e., the number of samples that contain a given food item), to summarize dietary data [79]. Twenty-two food items were identified in the feces of Siberian Cranes in the two winters, including 17 species, 3 genera, and 2 families (Fig. 3; Additional file 1: Table S2). Rice (RRA = 72.85%), lotus (12.01%), and *Polygonum criopolitanum* (11.99%) were the dominant food items in the feces (i.e., proportion > 10%) in winter 2017, and *Vallisneria* (56.12%) and *P. criopolitanum* (40.87%) were the dominant food items in winter 2018. Twenty-five food items were identified in the feces of White-naped Cranes, including 20 species, 3 genera, and 2 families. *Tulipa edulis* (37.37%), *Potentilla limprichtii* (18.69%), *P. criopolitanum* (16.52%), and *Carex* spp. (10.75%) were the dominant food items in the feces in winter 2017, and *P. criopolitanum* (84.41%) was the dominant food item in winter 2018. Twenty-two food items were identified in the feces of Hooded Cranes, including 18 species, 3 genera, and 1 family. *P. limprichtii* (65.36%) and *Carex* (27.39%) were the dominant food items in the feces in winter 2017, and *P. criopolitanum* (88.76%) was the dominant food item in winter 2018. Twenty-two food items were identified in the feces of Eurasian Cranes, including 17 species, 3 genera, and 2 families. Rice (61.20%) and *P. limprichtii* (23.94%) were the dominant food items in the feces in winter 2017, and *P. criopolitanum* (55.77%) and rice (27.40%) were the dominant food items in winter 2018. Diet compositions of Siberian Cranes (Pearson correlation = 0.05, *P* = 0.77), White-naped Cranes (Pearson correlation = 0.30, *P* = 0.08) and Hooded Cranes (Pearson correlation = − 0.04, *P* = 0.83) were not correlated between winter 2017 and winter 2018, suggesting great interannual variations in food sources. Diet composition of Eurasian Cranes was correlated (Pearson correlation = 0.40, *P* = 0.02) between the two winters, suggesting limited interannual variation in food sources.

*Vallisneria* tubers were an important food source for the four crane species, especially Siberian Cranes, in winter 2018. The proportion of *Vallisneria* was 56.12, 5.12, 8.50, and 7.34% in the feces of Siberian Cranes, White-naped Cranes, Hooded Cranes, and Eurasian Cranes, respectively, in winter 2018 (Fig. 3; Additional file 1: Table S2). In winter 2017, when *Vallisneria* tuber abundance was low, the proportion of *Vallisneria* was less than 1% in the feces of the four crane species.

Domesticated species identified in crane feces included rice and lotus. The proportions of domesticated species in the feces of Siberian Cranes (84.85%) and White-naped Cranes (15.09%) were high in winter 2017, but declined to 0.00% in winter 2018 (Fig. 3; Additional file 1: Table S2). Domesticated species occupied a small proportion in the feces of Hooded Cranes in winter 2017 (1.30%), but declined to 0.00% in winter 2018. The proportions of domesticated species in the feces of Eurasian Cranes were high in both winters, with 61.20% in winter 2017 and 27.40% in winter 2018.

### Dietary niche breadth and overlap among crane species

The numbers of food items, dietary niche breadth and Shannon-Wiener diversity values for Siberian Cranes and Eurasian Cranes in winter 2017 were similar to those in winter 2018 (Table 2). Values of all three parameters for White-naped Cranes and Hooded Cranes were higher in winter 2017 than in winter 2018. Dietary niche breadth of the four crane species was < 0.20 in both winters, indicating low niche breadth.

**Table 2:** Number of food items, dietary niche breadth, and Shannon-Wiener diversity values for Siberian Cranes, White-naped Cranes, Hooded Cranes, and Eurasian Cranes at Poyang Lake in the winters of 2017 and 2018

	Number of foods		Dietary niche breadth		Shannon-Wiener diversity	
	2017	2018	2017	2018	2017	2018
Siberian Crane	16	17	0.05	0.07	0.91	0.85
White-naped Crane	18	15	0.20	0.03	1.69	0.59
Hooded Crane	19	13	0.05	0.02	0.96	0.43
Eurasian Crane	18	19	0.08	0.09	1.10	1.21

**Fig. 4:**
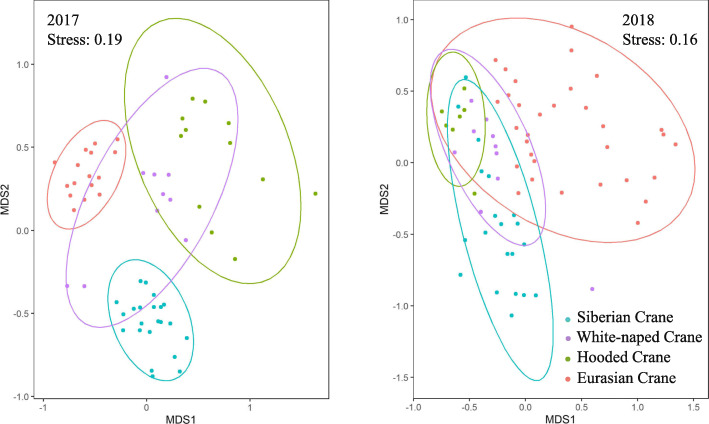
The results of nonmetric multidimensional scaling ordination (NMDS) based on Bray-Curtis dissimilarity. The colors of the circles correspond to the crane species: blue: Siberian Crane; purple: White-naped Crane; green: Hooded Crane; and red: Eurasian Crane

Dietary niche overlap among crane species ranged from 0.02 to 0.49 in winter 2017, but increased to 0.62 to 0.99 in winter 2018 (Table 3). Nonmetric multidimensional scaling (NMDS) analysis also indicated overlap increases in winter 2018 (Fig. 4). No overlap values were lower than expected by chance in the two winters. The overlap values were not higher than expected by chance in winter 2017. However, all values showed a high degree of overlap in winter 2018 [P (Obs > = null) < 0.05]. Overlap between White-naped Cranes and Hooded Cranes was the highest among the four crane species in both winters.

**Table 3:** The observed dietary niche overlap among the four crane species at Poyang Lake in the winters of 2017 and 2018. P (Obs < = null) and P (Obs > = null) indicate the tail probabilities for the observed index, which were lower and higher than the histogram of the simulated index, respectively. ^*^ represents *P* < 0.05, and ^**^ represents *P* < 0.01

Winter		Observed niche overlap index	P (Obs < = null)	P (Obs > = null)
2017	Siberian vs. White-naped	0.21	0.819	0.181
	Siberian vs. Hooded	0.02	0.703	0.297
	Siberian vs. Eurasian	0.16	0.900	0.100
	White-naped vs. Hooded	0.49	0.952	0.048
	White-naped vs. Eurasian	0.35	0.895	0.106
	Hooded vs. Eurasian	0.35	0.919	0.081
2018	Siberian vs. White-naped	0.63	0.952	0.048^*^
	Siberian vs. Hooded	0.66	0.952	0.048^*^
	Siberian vs. Eurasian	0.62	0.950	0.049^*^
	White-naped vs. Hooded	0.99	0.997	0.003^**^
	White-naped vs. Eurasian	0.89	0.987	0.013^*^
	Hooded vs. Eurasian	0.90	0.998	0.002^**^

## Discussion

### Diet composition

To the best of our knowledge, our study is the first to explore crane diets using metabarcoding techniques. Almost all previous studies have relied on traditional direct observation or microhistological methods. By using metabarcoding technology, we were able to elucidate crane diets at greater detail than previously known. The improved knowledge might be due to the ability of metabarcoding to provide a more comprehensive perspective and more details on food sources than traditional methods [66, 67, 80]. Metabarcoding is able to detect a variety of smaller and softer bodied food items that are overlooked by microhistological methods [67, 80]. It also enables the identification of food items at a higher taxonomic resolution [69, 81]. However, we acknowledge that our study has two limitations. First, we used fecal samples as they are non-invasive. Nevertheless, fecal analysis is subjected to the biases caused by the differential digestive rates of food items [82, 83]. Soft foods such as insect larvae tend to be under-represented in fecal samples because they are digested quickly and remain in gastro-intestinal tract for short periods, while hard foods such as mollusks tend to be over-represented because they break down slowly and remain in gastro-intestinal tracts longer. Thus, food proportions in the feces may not correlate with food proportions ingested by cranes. In addition, some materials identified in the feces may be incidentally consumed by cranes and not really be used as food. Second, our study only considered the plant component of cranes’ diets as plants were suggested to be their principal food types at Poyang Lake. Animal foods, consumed by the four crane species, but to a much lesser extent than plant foods [84–87], were not considered in our study.

The Siberian Crane is regarded as the most aquatic of all cranes, using wetlands for nesting, feeding and roosting [49, 88]. The high dependence of the species on aquatic habitats was suggested to make it vulnerable to impacts of habitat degradation and to lead its classification as Critically Endangered [49]. At Poyang Lake, Siberian Cranes traditionally fed on tubers of *Vallisneria*, the dominant submerged macrophytes, in shallow waters and mudflats [89, 90]. In winter 2010, due to a flood-induced *Vallisneria* tuber collapse, they were observed foraging for *P. limprichtii* taproots and *T. edulis* bulbs in grasslands for the first time [38, 44]. In the winters from 2015 to 2017, the diets of Siberian Cranes changed again, with thousands of cranes feeding on rice seeds and lotus rhizomes in agricultural habitats [61, 62, 64].

Our study identified 22 plant items in the feces of Siberian Cranes. These plant items were obtained from shallow waters, grasslands, and agricultural fields. Four plant items with yearly RRA > 10% were identified in the feces: *Vallisneria* tuber, *P. criopolitanum* rhizomes, rice seeds, and lotus rhizomes (the vegetative portions consumed by cranes were based on our field observation). Siberian Cranes’ diet compositions showed substantial interannual variation. When the abundance of *Vallisneria* tubers was low (i.e., winter 2017), Siberian Cranes mainly fed on rice seeds, lotus rhizomes, and *P. criopolitanum* rhizomes in agricultural fields and grasslands. When the abundance of *Vallisneria* tubers was high (i.e., winter 2018), they mainly fed on *Vallisneria* tubers and *P. criopolitanum* rhizomes in shallow waters and grasslands. Our study, in combination with previous studies, indicates that the diets of Siberian Cranes have changed greatly since winter 2010 [38, 44, 62]. They have gradually broadened their dietary niches, which has probably been driven by the degradation of submerged plants [38, 44, 57].

Similar to Siberian Cranes, a diet shift was also observed in White-naped Cranes. Historically, White-naped Cranes only fed on submerged plants in shallow waters and mudflats of Poyang Lake [91]. Then, driven by the decline of submerged plants, they primarily fed at grasslands and mudflats [87, 91], consuming *Vallisneria* tuber, *T. edulis*, *P. limprichtii*, and *Ranunculus polii* [55, 87]. Our results indicate that, in addition to *T. edulis* and *P. limprichtii* that had previously been reported, the proportions of *P. criopolitanum* and *Carex* were also high in the feces. Hooded Cranes were suggested to mainly feed on the grassland plant *P. limprichtii* through direct observation [86]. Our study indicates that the grassland plants *P. criopolitanum* and *Carex* also occupied high proportions in the feces. The dominant food items in the feces of White-naped Cranes and Hooded Cranes varied greatly between the two winters. The variation in dietary composition indicated that the diversity and composition of the grassland plant community might have changed greatly between the two winters. The dietary niche overlap between White-naped Cranes and Hooded Cranes was the highest among the four crane species, aligning with their level of habitat niche overlap, which was also the highest among the species [92]. The high dietary and habitat niche overlaps suggest high competition potential between the two crane species.

The Eurasian Crane is a habitat generalist. It is known to feed in a variety of habitats including grasslands, mudflats, shallow waters, and agricultural fields [88, 92]. Its diet at Poyang Lake has not been documented previously. Our study indicates that *P. criopolitanum*, *P. limprichtii*, and rice were the dominant food items in the feces of Eurasian Cranes. The number of plant items consumed, dietary niche breadth, and Shannon-Wiener measures were the highest among the four crane species, supporting its generalist foraging strategy. In contrast to the three crane species above, Eurasian Cranes’ diet compositions were similar between the two winters. Rice and grassland plants occupied high proportions in the feces in both winters.

### Important food resources for cranes

*Vallisneria* tubers have been acknowledged as important food sources for cranes, especially Siberian Cranes and White-naped Cranes [55, 56]. Our study confirms the importance of *Vallisneria* tubers in the diet of Siberian Cranes. It was the most common food source in the feces of Siberian Cranes in winter 2018, when its abundance was high. *Vallisneria* tuber proportions in the feces of the three other crane species were not as high as that in the feces of Siberian Cranes, but it still occupied 5–9% of the total foods in winter 2018.

To our surprise, *P. criopolitanum* was found to be the first dominant food in the feces of White-naped Cranes, Hooded Cranes, and Eurasian Cranes, and the second dominant food in the feces of Siberian Cranes in winter 2018. In winter 2017, the proportions of *P. criopolitanum* were < 17% in the feces of Siberian Cranes and White-naped Cranes, and were < 3% in the feces of Hooded Cranes and Eurasian Cranes. The higher proportions of *P. criopolitanum* in winter 2018 than winter 2017 indicate that the abundance and availability of *P. criopolitanum* was much higher in winter 2018. *P. criopolitanum* is an annual grassland plant. It is sensitive to hydrological conditions and can only survive in a relatively narrow range of water depths and percent time inundated [93]. Due to the sensitivity, its distribution range and abundance showed high interannual variation [94, 95]. Thus, it is likely that the abundance of *P. criopolitanum* was higher in winter 2018 than in winter 2017.

The importance of *P. criopolitanum* as a food source for the cranes at Poyang Lake has not been documented previously. It has only been suggested to be a major food source of Hooded Cranes at Shengjin Lake, another lake in the middle and lower Yangtze River floodplain [86, 96]. *P. criopolitanum* is a dominant wetland plant at Poyang Lake [93, 97]. The coverage percentage of this plant is the third highest among grassland plants, accounting for 12% of the total areas of grasslands [97]. This plant species is usually distributed at elevations of 9–12 m above sea level near mudflats and shallow waters [97], which are frequently used by cranes. Its rhizome, with a shallow burial depth, is high in energy and nutritional content and low in crude fibre [38, 86]. All these characteristics make *P. criopolitanum* rhizome a potentially important food source for cranes. We frequently observed cranes dig and feed on rhizomes of *P. criopolitanum* and leave aboveground parts in the fields. Therefore, we believe that *P. criopolitanum* was used as food, not incidentally consumed by cranes. However, we need to be caution that high proportion of *P. criopolitanum* in the feces does not mean high proportion in the diets. More work on the importance of *P. criopolitanum* in the diets of cranes is needed.

### The roles of agricultural fields in crane protection

With the loss and degradation of natural wetlands, the roles of artificial wetlands in waterbird protection have been under intensive debate [98, 99]. Although agricultural fields cannot completely replace the function of natural wetlands as waterbird habitats [100–102], agricultural fields might provide alternative or complementary habitats for waterbirds in all life stages [38, 103, 104]. The proportions of domesticated species in the feces of Siberian Cranes varied considerably between years. In winter 2017, when the abundance of *Vallisneria* tubers was low, the proportion of *Vallisneria* in Siberian Cranes’ feces was only 0.95%. Siberian Cranes may not have found enough foods in the shallow waters and mudflats and were forced to search for rice seeds and lotus rhizomes in agricultural fields. In winter 2018, when the abundance of *Vallisneria* tubers rebounded, Siberian Cranes moved back to shallow waters and mudflats, and a large proportion of their traditional food *Vallisneria* tubers, and also *P. criopolitanum* were detected in the feces. A similar abandonment of tuber foraging occurred following the tuber collapse in 2010 and then resumed in the next winter when tubers rebounded in 2011 [38, 44]. The diet and habitat changes indicate that agricultural habitats were important refuges for Siberian Cranes, providing important food resources for them to survive through winters with few *Vallisneria* tubers. They also indicate that Siberian Cranes prefer natural wetlands to agricultural fields [38].

Similar to Siberian Cranes, White-naped Cranes and Hooded Cranes consumed rice seeds and lotus rhizomes in winter 2017 but did not consume any in winter 2018. The changes in the proportions suggest that the abundance of food resources in natural wetlands might have been low in winter 2017. The deficient food resources might have been *Vallisneria* tubers, but might also have been grassland plants, the primary food sources of these two crane species. Under natural food shortage conditions (i.e., winter 2017), White-naped Cranes and Hooded Cranes utilized agricultural fields. When the abundance of *Vallisneria* tubers and probably grassland plants were high (i.e., winter 2018), the two crane species returned to their natural habitats. Therefore, for White-naped Cranes and Hooded Cranes, agricultural fields were important alternative foraging habitats used as buffers against starvation during periods of natural food shortages. The return of the two crane species to natural wetlands in winter 2018 further emphasizes the superior quality of natural habitats over agricultural fields as foraging habitats for cranes [38].

The proportions of rice seeds in the feces of Eurasian Cranes were high in the two winters, though the proportion in winter 2017 was twice as high as that in winter 2018. Therefore, agricultural fields appeared to be consistently important as foraging habitats for Eurasian Cranes. The frequent use of agricultural fields by Eurasian Cranes has been documented widely [49, 105, 106].

Domesticated species generally contain high energy and nutritional content and are of superior quality compared to natural plants [86, 104]. Farmland foraging has been suggested to contribute to increase in the abundance of some goose [107], egret [108] and wader [109] populations in Japan, Korea, Europe, and North America. However, cranes generally avoid human disturbance when select foraging sites [110, 111]. Human disturbance in agricultural fields around Poyang Lake is considerably high due to intensive agricultural activity [112]. High human disturbance was suggested to be a major reason that wintering geese were almost confined within natural wetlands and hesitated to exploit the riches of the modern agricultural habitats at Poyang Lake [112, 113]. High human disturbance also resulted in Siberian Cranes spending twice as much time alerting in agricultural fields than they did in natural habitats [114]. The high level of human disturbance in agricultural fields might contribute to the preference for natural wetlands by Siberian Cranes, White-naped Cranes and Hooded Cranes at Poyang Lake. Moreover, although agriculture fields contain energy-rich crops, wetlands contain diverse food resources, especially protein-rich invertebrates, which might better satisfy dietary needs of cranes [110, 115]. Taken together, security and diverse food resources might have attracted cranes to select wetlands as foraging sites.

Submerged macrophytes at Poyang Lake have degraded in recent decades [57–60], and *Vallisneria* tuber disappearance is expected to occur more frequently under future climate change [57, 60]. Cranes may increasingly depend upon agricultural fields around Poyang Lake, which may, in turn, cause significant crop damage and economic losses. This is especially true on agricultural fields surrounding protected wetlands where waterbirds concentrate in high numbers [116, 117]. At Poyang Lake, cranes mainly fed on post-harvest remains of rice seeds and lotus rhizomes; thus, crop damage and economic loss were limited. However, we observed some farmers scared cranes away from their agricultural fields. There were more than 14 million free-ranging poultry raised at agricultural fields around Poyang Lake [118], where they gleaned waste grain residues in stubble fields. Crane feeding would reduce the food abundance of the free-ranging domestic poultry; thus, farmers drove cranes away. To reduce human disturbance, environmental education is needed because farmers are more likely to protect cranes if they understand the endangered degree and the ecological significance of cranes.

### Effects of tuber collapse on dietary niche breadth and overlap among crane species

*Vallisneria* tuber is an important food source for Siberian Cranes (89, 90, this study); thus, variation in its abundance might influence Siberian Cranes’ dietary niche breadth. The numbers of food items, dietary niche breadth and Shannon-Wiener measures of Siberian Cranes were similar between winter 2017 and winter 2018. This is inconsistent with the contention that during seasons with a low abundance of dominant food, the dietary niche may broaden as consumers relying on insufficient preferred food items are forced to add less profitable resources to their diets [26, 119, 120]. In winter 2017, with low *Vallisneria* tuber abundance, Siberian Cranes left shallow waters and mudflats and moved to rice paddies and lotus ponds [61, 62, 64]. Their foods changed from *Vallisneria* tuber to rice seeds and lotus rhizomes almost completely. As the energy, protein and fat contents of crops are generally as good as or superior to natural foods and are often present in agricultural fields in far greater abundance [86, 104], Siberian Cranes may gain enough energy through feeding on crops and do not need to add many other food sources. Moreover, the Siberian Crane is a dietary specialist [89, 90]. Poyang Lake has a relatively simple plant community composition, with *Vallisneria* and *Hydrilla verticillate* being the dominant submerged macrophytes [59] and *Carex* being the dominant grassland plants [121]. Therefore, there may be few options for Siberian Cranes, which might lead to the failure of this species to widen its dietary niche breadth.

In contrast to Siberian Cranes, the numbers of food items, dietary niche breadths and Shannon-Wiener measures of White-naped Cranes and Hooded Cranes were higher in winter 2017 than in winter 2018. *P. criopolitanum* was the predominant food item in the feces of the two crane species in winter 2018, and the proportions of *Vallisneria* tubers were also high. The abundances of *Vallisneria* tubers and probably *P. criopolitanum* at Poyang Lake declined greatly in winter 2017. When there is a short-fall in food availability, animals may optimize foraging behavior and increase their dietary breath [122, 123]. The short-fall in the abundances of *P. criopolitanum* and *Vallisneria* tubers in winter 2017 might have driven the two crane species to add less profitable grassland plants in their diets. Although the two cranes also consumed high-quality rice seeds and lotus rhizomes in winter 2017, their proportions were low in the feces and these foods might be unable to meet the energy requirements of the two crane species. The niche expansion of the two crane species during periods of preferred food shortage was in agreement with the basic prediction of optimal foraging theory [22, 23]. It is also consistent with a commonly observed fact that energy-maximizing models work better when animals food-limited [124].

The dietary niche overlaps among the crane species were low in winter 2017, with the four crane species diverging in their principal food items. However, in winter 2018, the niche overlaps were significantly higher than expected by chance. They had similar diet compositions, with *P. criopolitanum* rhizomes and *Vallisneria* tubers being the principal food items in the feces. The increased overlap might have been caused by the high abundances of the two plant species in winter 2018, which allowed the four crane species to share the same food items with low interspecific competition. Our study is consistent with previous research that suggested that among-species dietary niches tended to overlap more during seasons of high food abundance [32, 33, 125, 126]. Greater resource availability could promote an increase in species utilization of similar resources and reduce competitive interactions between species [32, 34, 126]. By contrast, during seasons with low food abundance, species may partition limited food resources to decrease interspecific competition, leading to a decrease in niche overlap [126, 127]. It has been suggested that diet partitioning facilitates the coexistence of many closely related species [7, 128]. The interannual variation in niche overlap observed in our study suggests that diet partitioning may be restricted to specific temporal cases. In seasons or years with abundant food sources, interspecific competition may be relaxed and niche overlap could be high.

## Conclusions

In summary, by using metabarcoding technology, our study gained new insights into cranes’ diets that are essential for informing habitat management to improve the availability of foraging resources. *Vallisneria* tubers was confirmed as an important food source of cranes. Surprisingly, *P. criopolitanum* also occupied high proportions in the feces of cranes and may also be important. Our study emphasizes the superior quality of natural wetlands compared to agricultural fields as foraging habitats for cranes. The recent degradation of submerged macrophytes at Poyang Lake potentially threatens the survival of cranes, especially Siberian Cranes. Therefore, it is urgent to reverse the broad decline of submerged macrophytes and provide important foraging habitats for cranes during years of tuber collapses. The abundances of *Vallisneria* tubers, and probably *P. criopolitanum*, at Poyang Lake greatly influenced the dietary niche width and overlaps among crane species. Cranes exhibited low dietary niche overlap during periods of preferred food shortage but significantly high overlap during periods when preferred foods were plentiful. While high niche overlap is often interpreted as intense interspecific competition, our study highlights the importance of taking food abundance into account.

## Materials and methods

### Study areas

Poyang Lake, the largest freshwater lake in China, is one of the most important wintering areas for waterbirds along the East Asian-Australasian Flyway (Fig. 1) [51, 129]. It is characterized by dramatic seasonal hydrological fluctuations, with an average change of 9.24 m between the summer high and winter low water levels [130]. During summer, the inundation area is > 2500 km^2^ [131]. In autumn and winter, the water level drops, causing the inundation area to shrink to < 1000 km^2^ [131]. Poyang Lake contains 102 sublakes, which are inundated and integrated with the main body of Poyang Lake during summer and are isolated during winter [132]. As the water level drops, the sublakes appear, and large areas of grasslands, mudflats, and shallow water areas are exposed, providing winter foraging and roosting areas for approximately 420,000 waterbirds from approximately 111 species [133, 134].

### *Vallisneria* tuber survey

The density and biomass of *Vallisneria* tubers were surveyed at three sublakes (Dahuchi, Shahu, and Changhuchi; Fig. 1) of Poyang Lake in late October and early November of 2017 and 2018 before large numbers of wintering waterbirds arrived. We set 76, 183, and 36 sampling plots uniformly located at Dahuchi, Shahu, and Changhuchi, respectively. *Vallisneria* tubers were collected from two quadrats in each plot with a locally made steel grab sampler. The sampler has two long handles. At the end of the handles, there are two scoops facing each other. The sampler was inserted into the substrate and the scoops brought together to collect about 15 cm long × 13 cm wide × 30 cm high sample of substrate. Tubers were subsequently cleaned and counted. The dry weight of tubers in each quadrat was weighed after oven-drying at 80 °C until constant weight. The tuber density and biomass in each plot was calculated by dividing total tuber number and tuber mass across two quadrats by quadrat area, respectively. We used t-test to explore whether the tuber density and biomass in the winters of 2017 and 2018 significantly deviated from the historical average values in the winters from 1999 to 2016 [57]. The historical tuber density and biomass data were collected by the Poyang Lake National Nature Reserve at three sublakes (Dahuchi, Shahu, and Meixihu) [57]. R 3.6.0 was used to do the t-test analyses.

### Fecal sample collection

Fecal samples were collected at Poyang Lake in the winters of 2017 and 2018. We first investigated the distributions of cranes. When we saw foraging cranes, we waited until they left to avoid disturbing them. Soon after they left, we went to the foraging sites to collect fresh feces (i.e., feces with wet surface) with sterilized tweezers. To minimize the probability of multiple samples from the same individual, all collected samples were separated by at least 5 m. We stored the samples in liquid nitrogen in the field and transferred them to a − 80 °C refrigerator for long-term storage in the laboratory. To ensure that the sources of the feces were known, we tried to collect samples from monospecific flocks. For samples collected from mixed-species flocks, we used DNA analysis to identify the species that deposited the fecal samples. Plants were also collected in the field to construct a DNA barcoding reference database.

### Species identification

Because of the similar morphologies of the feces of the four crane species, we used DNA analysis to identify the species that deposited the fecal samples collected from mixed-species flocks. For the genetic species assignment of each fecal sample, we scraped the external surface, which contain DNA from the host species as a result of the sloughing of cells from the digestive tract. DNA was extracted with the QIAamp Power Fecal DNA Kit (Qiagen, USA) according to the manufacturer’s guidelines. For each round of DNA extraction, negative controls (i.e., extraction without feces) were included to monitor for possible contamination. We amplified the mitochondrial DNA D-loop fragment with the primer pair DL02F (5′-3′ GCGGCCCGAAAAGCCGCTG) and DL02R (5′-3′ GCCCTGACATAGGAACCAGAGGCGC) [135]. PCR amplifications were carried out in a total volume of 25 μl containing 12.5 μl PCR mix (Tiangen, Beijing, China), 1 μl DNA, 1 μl each primer, and 9.5 μl H_2_O. The reaction conditions were as follows: denaturation at 94 °C for 4 min, followed by 34 cycles at 94 °C for 48 s, 56 °C for 48 s, and 72 °C for 1 min, and a final 10 min at 72 °C. We checked for the presence of a PCR product of suitable length by electrophoresis on a 1% agarose gel. Sequencing was carried out on an ABI 3730xl analyzer by Sangon Biotech (Shanghai, China). The complete sequences were assembled using Seqman II (DNASTAR) and compared visually to the original chromatograms to avoid reading errors. The taxonomic assignment was conducted by searching against the nucleotide database of GenBank using Web BLAST.

### Construction of a barcode reference database

We downloaded the *trn*L sequences of 48 plant species commonly seen at Poyang Lake from GenBank. For the plant species not included in GenBank, we sequenced *trn*L by ourselves. Total DNA was extracted using the hexadecyltrimethylammonium bromide (CTAB) protocol. We used primer pair *c* (5′-3′ CGAAATCGGTAGACGCTACG) and *h* (5′-3′ CCATTGAGTCTCTGCACCTATC) to amplify an approximately 150 bp region of the chloroplast *trn*L intron [136]. This primer pair was chosen as it can amplify DNA from a wide range of wetland plants [70]. PCR amplifications were carried out in a total volume of 25 μl containing 12.5 μl PCR mix (Tiangen, Beijing, China), 1 μl DNA, 1 μl each primer, and 9.5 μl H_2_O. The reaction conditions were as follows: denaturation at 94 °C for 4 min, followed by 35 cycles at 94 °C for 30 s, 56 °C for 30 s, and 72 °C for 45 s, and a final 10 min at 72 °C. We used electrophoresis to check the PCR products, ABI 3730xl analyzer to perform sequencing, and Seqman II to assemble the sequences.

### DNA extraction and high-throughput sequencing of fecal samples

To uncover plant component consumed by cranes, a modified CTAB protocol [70] was used to extract the DNA from approximately 200 mg of the interior of each fecal sample. DNA extraction was conducted in a dedicated laboratory for analyses of DNA from samples with low DNA quality. For each batch of DNA extraction, negative controls were included to monitor for possible contamination. The primers *c* and *h* with a 6 bp tag added to 5′ ends of each primer were used to amplify *trn*L gene. The PCR amplification procedure was the same as barcode database construction. For each sample, a unique combination of tags in the forward and reverse primers was used so that sample-specific data can be recovered after sequencing. Three PCR replicates were performed for each sample to reduce amplification bias. The three replicates were pooled, and the DNA concentration and purity were monitored on a 1% agarose gel. Only samples with a clear band were processed further. Samples were purified using the SanPrep Column PCR Product Purification Kit (Sangon Biotech, Shanghai, China). The purified PCR products were pooled, taking into account of DNA concentration to obtain an approximately equal amount of amplicon DNA from each fecal sample. The pool of individually tagged amplicons was used to prepare a sequencing library using the TruSeq DNA PCR-Free Sample Preparation Kit (Illumina, USA) following manufacturer’s recommendations. The library was sequenced on an Illumina HiSeq 2500 platform, and 250 bp paired-end reads were generated. Library preparation and sequencing were conducted by Novogene (Beijing, China).

### Sequencing data analysis

After high-throughput sequencing, the paired-end reads were demultiplexed into sample-specific files based on their unique tags. The tags and primer sequences were subsequently trimmed. The paired-end reads were merged using FLASH 1.2.7 [137] and then filtered using QIIME 1.7.0 [138]. Sequences were dereplicated and clustered to OTUs using USEARCH 11.0.667 [139] at the similarity threshold of 97%. All OTUs were assigned to taxonomic units by referring to the local plant database using blast 2.2.31+, with thresholds of identity > 97% and e-value < 1.0 e^− 50^. If an OTU matched two or more taxa, it was assigned to a higher taxonomic level that included all taxa.

To evaluate the sufficiency of sequencing depth and sampling size, individual-, species-, and sample-based rarefaction curves were built using EstimateS 8.2 [140]. The curves were computed by randomly resampling sequences or samples and plotting these against the numbers of OTUs. The read abundance data were converted to RRA of each food item using the following equation [79]:

$$ \mathrm{RRA}=\frac{1}{\mathrm{S}}{\sum}_{\mathrm{K}=1}^{\mathrm{S}}\frac{{\mathrm{n}}_{\mathrm{i},\mathrm{k}}}{\sum_{\mathrm{i}=1}^{\mathrm{T}}{\mathrm{n}}_{\mathrm{i},\mathrm{k}}}\times 100\%, $$

where n_i,k_ is the number of sequences of food item i in sample k, T is the total number of food items, and S is the sampling size.

### Dietary niche breadth and overlap among crane species

To explore whether the cranes’ diets showed substantial interannual variation, we calculated Pearson correlations between diet compositions of each crane species in winter 2017 and winter 2018. The proportion of each food item in each sample was used as input file. The Shannon-Wiener diversity index (H′) was calculated to explore the diet diversity of each crane species. The following equations were used:

$$ {\mathrm{H}}^{\prime }=-\sum \limits_{\mathrm{i}=1}^{\mathrm{s}}{\mathrm{P}}_{\mathrm{i}}\ln {\mathrm{P}}_{\mathrm{i}}, $$

where P_i_ is the proportion of food item i out of all foods and S is the total number of food items. The dietary breadth (B) was measured using Levins’ index [141], according to the following formula:

$$ \mathrm{B}=1/{\sum}_{\mathrm{i}=1}^{\mathrm{s}}{\mathrm{P}}_{\mathrm{i}}^2, $$

Hurlbert’s formula [142] was applied to standardize the trophic niche measure, according to the following formula:

$$ {\mathrm{B}}_{\mathrm{a}}=\left(\mathrm{B}-1\right)/\left(\mathrm{S}-1\right), $$

where B is the Levins index of niche breadth and S is the total number of prey categories. B_a_ values range between 0 (minimum diet breadth) and 1 (maximum diet breadth).

The dietary overlap of each species pair (Q_jk_) was calculated using Pianka’s index [143], according to the following formula:

$$ {\mathrm{Q}}_{\mathrm{jk}}=\frac{\sum_{\mathrm{i}=1}^{\mathrm{s}}{\mathrm{P}}_{\mathrm{i}\mathrm{j}}{\mathrm{P}}_{\mathrm{i}\mathrm{k}}}{\sqrt{\sum_{\mathrm{i}=1}^{\mathrm{s}}{\mathrm{P}}_{\mathrm{i}\mathrm{j}}^2{\sum}_{\mathrm{i}=1}^{\mathrm{s}}{\mathrm{P}}_{\mathrm{i}\mathrm{k}}^2}} $$

where Q_jk_ is Pianka’s niche overlap index between species j and species k; P_ij_ is the proportion of resource i out of all resources used by species j; and P_ik_ is the proportion of resource i out of all resources used by species k. Q_jk_ ranges from 0, where two species have no food items in common, to 1, where there is complete overlap in resource use.

We tested whether the observed niche overlap differed from what would be expected by chance using the program EcoSim 1.00 [144]. Null models were calculated based on the randomization algorithm RA3 and 1000 simulated resource utilization matrices were generated to compare with observed resource utilization data.

Patterns of diet composition and overlap of the four crane species were visualized in two-dimensional space using the NMDS plots. We performed NMDS analysis based on the Bray-Curtis dissimilarity. The read abundance of each food item in each sample was used as the input file. The R package “Vegan” [145] was used for the NMDS analysis, and “ggplots” [146] was used to create graphics.

## Supplementary Information

**Additional file 1: Table S1.** Sampling site information. **Table S2.** Relative read abundance (RRA; %) of each food item in the diets of Siberian Cranes, White-naped Cranes, Hooded Cranes, and Eurasian Cranes in the winters of 2017 and 2018. **Figure S1.** Rarefaction curve of each crane species.

## Data Availability

The datasets used and/or analyzed during the current study are available from the corresponding authors on reasonable request.

## Abbreviations

NMDS: Nonmetric multidimensional scaling
OTU: Operational taxonomy units
RRA: Relative read abundance
