# Supplementary material for: Influences of submerged plant collapse on diet composition, breadth, and overlap among four crane species at Poyang Lake, China
Source: Front Zool. 2021 May 17;18:24. doi: 10.1186/s12983-021-00411-2 (PMC8130136; doi:10.1186/s12983-021-00411-2)

**Additional file 1**

Table S1 Sampling site information. Winter was defined as the year of December.

| Species | Winter | Date | Location | Longitude | Latitude | Sample size | Habitat type |
| --- | --- | --- | --- | --- | --- | --- | --- |
| Siberian Crane | 2017 | Nov. 22, 2017 | Wuxing Farmland, Nanchang | E116°15'44" | N28°46'48" | 4 | Rice paddy |
|  |  | Dec. 17, 2017 | Henghu Farmland, Nanchang | E116°38'8" | N29°8'11" | 2 | Rice paddy |
|  |  | Mar. 4, 2018 | Yongxiu County, Jiujiang | E115°54'49" | N29°5'20" | 3 | Rice paddy |
|  |  | Mar. 5, 2018 | Henghu Farmland, Nanchang | E116°2'49" | N28°59'14" | 2 | Rice paddy |
|  |  | Mar. 14, 2018 | Wuxing Farmland, Nanchang | — | — | 4 | Lotus pond |
|  |  | Mar. 15, 2018 | Zhugang Farmland, Nanchang | E116°10'7" | N28°55'15" | 3 | Rice paddy |
|  |  | Apr. 15, 2018 | Wuxing Farmland, Nanchang | E116°12'40" | N28°46'26" | 4 | Lotus pond |
|  | 2018 | Jan. 3, 2019 | Zhushi Lake, Jiujiang | E115°59'12" | N29°10'12" | 7 | Mudflat |
|  |  | Jan. 6, 2019 | Changhu Lake, Jiujiang | E115°59'40" | N29°8'4" | 7 | Mudflat |
|  |  | Jan. 13, 2019 | Zhushi Lake, Jiujiang | E115°58'32" | N29°10'45" | 6 | Mudflat |
| White-naped Crane | 2017 | Dec. 13, 2017 | Shahushan, Jiujiang | E115° 53'45" | N29°10'33" | 5 | Rice paddy |
|  |  | Mar. 16, 2018 | Sha Lake, Jiujiang | E115°55'1" | N29°11'44" | 5 | Grassland |
|  | 2018 | Dec. 8, 2018 | Bang Lake, Jiujiang | E115°55'12" | N29°13'28" | 10 | Grassland |
| Hooded Crane | 2017 | Jan. 18, 2018 | Hengfeng Farmland, Jiujiang | E115°51'25" | N29°8'4" | 4 | Rice paddy |
|  |  | Mar. 16, 2018 | Shahushan, Jiujiang | E115°53'41" | N29°11'24" | 8 | Grassland |
|  | 2018 | Jan. 3, 2019 | Meixi Lake, Jiujiang | E116°3'41" | N29°12'47" | 7 | Mudflat |
| Eurasian Crane | 2017 | Nov. 22, 2017 | Wuxing Farmland, Nanchang | E116°18'16" | N28°46'2" | 4 | Rice paddy |
|  |  | Dec. 8, 2018 | Wuxing Farmland, Nanchang | E116°17'36" | N28°47'14" | 3 | Rice paddy |
|  |  | Jan. 1, 2018 | Henghu Farmland, Nanchang | — | — | 2 | Rice paddy |
|  |  | Feb. 2, 2018 | Yongxiu County, Jiujiang | E115°53'27" | N29°4'55" | 3 | Rice paddy |
|  |  | Feb. 3, 2018 | Kangshan Farmland, Shangrao | E116°25'14" | N28°52'18" | 4 | Rice paddy |
|  | 2018 | Oct. 18, 2018 | Qinlan Lake, Nanchang | E116°12'7" | N28°34'58" | 5 | Grassland |
|  |  | Oct. 31, 2018 | Duchang County, Jiujiang | E116°17'5" | N29°10'47" | 7 | Grassland |
|  |  | Dec. 18, 2018 | Shahushan, Jiujiang | E115°53'36" | N29°10'23" | 4 | Rice paddy |
|  |  | Dec. 23, 2018 | Gongqingcheng, Jiujiang | E115°52'11" | N29°13'50" | 4 | Rice paddy |
|  |  | Dec. 25, 2018 | Yongxiu County, Jiujiang | E115°52'13" | N29°3'49" | 4 | Rice paddy |
|  |  | Jan. 17, 2019 | Bianyu Lake, Shangrao | E116°25'32" | N28°55'3" | 8 | Grassland |

Table S2 Relative read abundance (RRA; %) of each food item in the diets of Siberian Crane, White-naped Crane, Hooded Crane, and Eurasian Crane in the winters of 2017 and 2018. Bold font represents RRA > 10%.

| Food items | Siberian Crane | | White-naped Crane | | Hooded Crane | | Eurasian Crane | |
| --- | --- | --- | --- | --- | --- | --- | --- | --- |
|  | 2017 | 2018 | 2017 | 2018 | 2017 | 2018 | 2017 | 2018 |
| *Vallisneria* spp. | 0.952 | **56.120** | 0.257 | 5.120 | 0.064 | 8.500 | 0.012 | 7.340 |
| *Polygonum criopolitanum* | **11.985** | **40.870** | **16.518** | **84.410** | 0.644 | **88.760** | 2.573 | **55.770** |
| *Potentilla limprichtii* | 0.413 | 0.130 | **18.692** | 0.510 | **65.358** | 0.010 | **23.935** | 0.150 |
| *Amana edulis* | 0.332 | 0.000 | **37.368** | 0.000 | 3.031 | 0.000 | 0.145 | 0.000 |
| *Nelumbo nucifera* | **72.846** | 0.000 | 5.638 | 0.000 | 0.432 | 0.000 | 0.000 | 0.000 |
| *Oryza sativa* | **12.009** | 0.000 | 9.456 | 0.000 | 0.873 | 0.000 | **61.197** | **27.400** |
| *Carex* spp. | 0.624 | 0.540 | **10.753** | 0.060 | **27.389** | 0.000 | 0.581 | 0.050 |
| Poaceae | 0.000 | 1.190 | 0.001 | 9.240 | 0.011 | 0.090 | 0.000 | 0.050 |
| *Phalaris arundinacea* | 0.244 | 0.490 | 0.861 | 0.290 | 0.653 | 0.030 | 8.854 | 0.450 |
| *Alopecurus japonicus* | 0.069 | 0.010 | 0.104 | 0.000 | 0.146 | 2.380 | 2.320 | 4.060 |
| *Fimbristylis polytrichoides* | 0.450 | 0.000 | 0.233 | 0.020 | 1.001 | 0.000 | 0.213 | 4.260 |
| *Trapa incisa* | 0.000 | 0.470 | 0.000 | 0.030 | 0.186 | 0.000 | 0.000 | 0.000 |
| *Rumex* spp. | 0.000 | 0.010 | 0.000 | 0.000 | 0.000 | 0.000 | 0.004 | 0.340 |
| *Polygonum* spp. | 0.000 | 0.000 | 0.000 | 0.000 | 0.151 | 0.000 | 0.000 | 0.000 |
| *Astragalus sinicus* | 0.011 | 0.000 | 0.005 | 0.000 | 0.002 | 0.000 | 0.087 | 0.000 |
| *Poa annua* | 0.004 | 0.000 | 0.020 | 0.000 | 0.007 | 0.000 | 0.000 | 0.000 |
| *Cotula anthemoides* | 0.000 | 0.000 | 0.000 | 0.040 | 0.000 | 0.000 | 0.000 | 0.000 |
| *Erigeron annuus* | 0.000 | 0.000 | 0.014 | 0.000 | 0.000 | 0.000 | 0.010 | 0.000 |
| *Cardamine lyrata* | 0.000 | 0.010 | 0.000 | 0.050 | 0.000 | 0.080 | 0.000 | 0.010 |
| *Limosella aquatica* | 0.005 | 0.000 | 0.000 | 0.000 | 0.000 | 0.000 | 0.000 | 0.000 |
| *Miscanthus lutarioriparius* | 0.038 | 0.010 | 0.065 | 0.040 | 0.041 | 0.000 | 0.018 | 0.090 |
| *Soliva anthemifolia* | 0.000 | 0.080 | 0.000 | 0.040 | 0.000 | 0.140 | 0.000 | 0.010 |
| *Artemisia selengensis* | 0.000 | 0.000 | 0.006 | 0.070 | 0.000 | 0.000 | 0.000 | 0.000 |
| *Cynodon dactylon* | 0.000 | 0.000 | 0.001 | 0.000 | 0.004 | 0.000 | 0.045 | 0.000 |
| *Gratiola japonica* | 0.013 | 0.000 | 0.000 | 0.000 | 0.005 | 0.000 | 0.000 | 0.000 |
| Nymphaeaceae | 0.000 | 0.050 | 0.000 | 0.070 | 0.000 | 0.000 | 0.000 | 0.010 |
| *Eleocharis valleculosa* | 0.000 | 0.000 | 0.008 | 0.000 | 0.000 | 0.000 | 0.001 | 0.000 |
| *Galium trifidum* | 0.000 | 0.000 | 0.000 | 0.000 | 0.000 | 0.000 | 0.002 | 0.000 |
| *Nymphoides peltata* | 0.004 | 0.000 | 0.000 | 0.000 | 0.001 | 0.000 | 0.002 | 0.000 |

Figure S1 Rarefaction curve of each crane species. OTU is the abbreviation of operational taxonomy units.


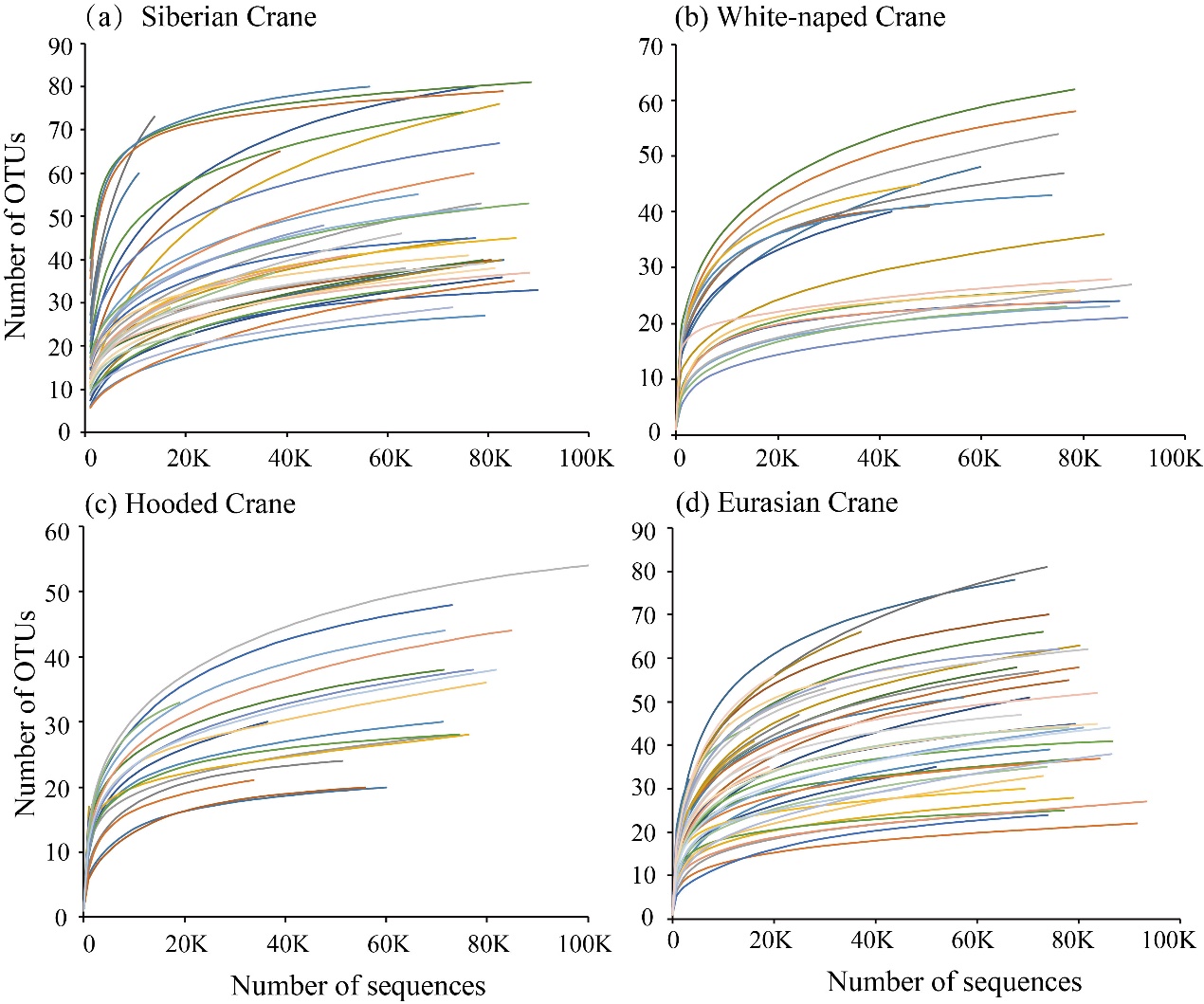

Supplement: Supplementary file 1 — Additional file 1: Table S1. Sampling site information. Table S2. Relative read abundance (RRA; %) of each food item in the diets of Siberian Cranes, White-naped Cranes, Hooded Cranes, and Eurasian Cranes in the winters of 2017 and 2018. Figure S1. Rarefaction curve of each crane species. [file 12983_2021_411_MOESM1_ESM.docx]
